# Base Promoted Intumescence of Phenols

**DOI:** 10.3390/polym12020261

**Published:** 2020-01-23

**Authors:** Yu Ji, Qiang Yao, Weihong Cao, Yueying Zhao

**Affiliations:** 1 Ningbo Key Laboratory of Polymer Materials, Ningbo Institute of Material Technology and Engineering, Chinese Academy of Sciences, Ningbo 315201, China; jiyu@nimte.ac.cn (Y.J.); caoweihong@nimte.ac.cn (W.C.); zhaoyueying@nimte.ac.cn (Y.Z.); 2 University of Chinese Academy of Sciences, Beijing 100049, China; 3 Key Laboratory of Bio-based Polymeric Materials Technology and Application of Zhejiang Province, Ningbo 315201, China

**Keywords:** alkali, intumescence, substituent, hydroarylation, phenolate

## Abstract

The intumescent process of sodium (substituted) phenolates has been studied. The generation of hydrogen radical via a homolytic cleavage of the Ar–H bond and the subsequent hydroarylation of phenolates to cyclohexadienes along with cyclization and elimination reactions of cyclohexadienes are critical steps in the base promoted intumescence of phenols. The substituents show great influence on the intumescence of phenolates. Phenolates substituted with a weak electron donating group enable intumescence while those with an electron withdrawing group or strong electron donating group suppresses intumescence. This distinction can be justified by both electronic and steric effects of substituents on the generation of hydrogen radical and the degree of hydroarylation.

## 1. Introduction

Intumescence is the formation of expanded carbonaceous char. It has been a widely-used technique in the flame retardancy of polymeric materials [1,2,3,4]. Due to its smoke suppression and low toxicity [5,6], considerable efforts have been devoted to understanding the chemical and physical processes involved in the development of foamed cellular charred layer by intumescent flame retardants (IFRs) [7,8,9,10]. It has been generally believed that three ingredients are necessary for IFRs to achieve good intumescence, i.e., an acid source, a char forming agent, and a blowing agent [11,12]. Among these three components, the acid is especially important. It not only acts as a catalyst for the carbonization of charring agents through esterification, elimination, and cyclization reactions [13,14,15,16,17,18], but also functions as bridges, together with aliphatic structures, to link the hard polyaromatic stacks [19,20]. These bridges act as soft segments to regulate the viscosity of intumescent materials and hence are the key to good intumescence [21]. In these aspects, the acid plays an indispensable role in the traditional intumescent process.

However, we recently discovered that alkali promotes the intumescence of phenols [22]. This is drastically different from the acid based intumescence. Since alkali hydroxides are regenerated in the intumescent process of phenolates, this potentially permits us to develop a catalytic intumescent flame retardant based on polyphenols and a catalytic amount of bases. Nevertheless, in order to achieve this goal, an understanding of the chemistry and the physical process involved in the alkali promoted intumescence of phenols is needed.

Further, alkalis have been well known to catalyze the formation of highly microporous carbons during pyrolysis and gasification of coals and biomass [23,24]. Although many accounts, mainly focusing on the reduction of alkali salts and their influence on the primary reactions such as esterification and depolymerization, have been offered to justify this alkali’s effect [25,26,27,28], considering the ubiquitous presence of phenolic structures and alkalis in coals and heavily aromatic biomass [28,29], alkali promoted intumescence of phenols might be the key to understand the dilation of char produced in the carbonization of coals and biomass.

In this work, the intumescent process of (substituted) phenolates has been studied. It has been found that the generation of the hydrogen radical is the basis for the intumescence and that the electronic nature and the steric factor of substituents decisively influence the intumescent process of phenolates. Detailed chemistry to polyarenes and polycyclic aromatic hydrocarbons (PAHs), which are essential components of intumescence has also been established. Our work will not only be constructive to stimulate the development of catalytic intumescent flame retardants, but also help comprehend the substantial effects of alkali on the quality of char produced from the carbonization of biomass and coals [28].

## 2. Experimental

### 2.1. Materials

Sodium phenolate was purchased from Energy Chemical Co. Ltd. (Shanghai, China). Sodium hydroxide, *p*-ethylphenol, *p*-cresol, *m*-cresol, 2,2′-biphenol, 4,4′-biphenol, and hydroquinone were bought from Sinopharm Chemical Reagent Co. Ltd. (Shanghai, China). Sodium salicylate and *p*-phenoxyphenol were obtained from Aladdin Co. (Shanghai, China). All materials were used without further purification.

### 2.2. Preparations of Sodium Phenolates

Sodium salts of *p*-ethylphenol, *p*-cresol, *m*-cresol, *p*-phenoxyphenol, salicylate, 2,2′-biphenol, 4,4′-biphenol, and hydroquinone were prepared by neutralization with aqueous sodium hydroxide at a 1/1 mole ratio (mono-OH/NaOH) or a 1/2 mole ratio (di-OH/NaOH). The neutralized products were dried to a constant weight in a vacuum oven at 110 °C.

### 2.3. Thermal Treatment

Continuous heating treatment: samples of about 15 mg each of sodium phenolate, sodium *p*-ethylphenolate, sodium *p*-cresolate, sodium *p*-phenoxyphenolate, di-sodium biphenolates, di-sodium hydroquinolate, and mono-/di- sodium salicylate were heated in a Q500 thermogravimetric instrument (TA Instrument, New Castle, DE, USA) at a heating rate of 10 °C/min under nitrogen (60 mL/min). Residues were collected during the major degradation step for analysis.

Isothermal treatment of sodium phenolate: a series of sodium phenolate of about 15 mg each was heated in a Q500 thermogravimetric instrument from 50 to 495 °C at a rate of 10 °C/min under a nitrogen flow of 60 mL/min and was then kept heated at that temperature for a different duration (0, 2.5, 4.5, 5.2, 5.4, 5.5, 5.6, 6, and 8 min) until the residue was almost insoluble in methanol. The collected residues were subjected to analysis.

### 2.4. Characterizations

Fourier transform infrared spectra (FTIR) of the residues obtained from thermal treatment of sodium salts were recorded on a Cary 660 FTIR spectrometer (Agilent Technologies, Santa clara, CA, USA) interfaced to a GladiATR (Pike Technologies, Fitchburg, WI, USA) with a diamond crystal at 4 cm^−1^ resolution.

Nuclear magnetic resonance (NMR) of residues was performed on a Bruker 400 AVANCE spectrometer (Bruker, Karlsruhe, Germany). ^1^H and ^13^C composite pulse decoupled (^13^C CPD) NMR measurements were run at frequencies of 400 MHz and 100 MHz respectively. Methanol-*d*4 was used as a solvent. Proton chemical shifts were referenced to tetramethylsilane (TMS).

The molecular weight measurement of residues of sodium phenolate after thermal treatments was carried out by a Quadra pole-time of flight mass spectrometer (Q-TOF) and gel permeation chromatography (GPC) on a TripleTOF 4600 mass spectrometer (AB Sciex, Framingham, MA, USA) and an HLC-8320 GPC (TOSOH Bioscience, Tokyo, Japan) respectively. The atmospheric pressure chemical ionization (APCI) mode in Q-TOF was selected according to the polarity of the products. The negative ion mode was used to analyze the fragment ions. The mass range for the mass investigation was set at m/z = 50–1000 Da. The ion spray voltages were set to 4500 V. The declustering potential was 80 V. The temperature of vaporizer was 500 °C. The injection rate was 5 μL/min. In GPC, about 10 mg of sample was dissolved in 2 mL of chromatographically pure dimethyl formamide (DMF). The injection volume was 80 μL and the flow rate of the mobile phase (DMF) was 0.6 mL/min. The temperature of the column was set to 40 °C.

## 3. Results and Discussion

### 3.1. Intumescent Process of Sodium Phenolate

#### 3.1.1. Appearance 

Sodium phenolate has been observed to generate intumescence during a continuous heating treatment [22]. However, due to the quick mass loss, it was inconvenient to study the intumescent process of sodium phenolate. Consequently, an isothermal treatment was carried out in order to separate different stages in the intumescent process.

**Figure 1:**
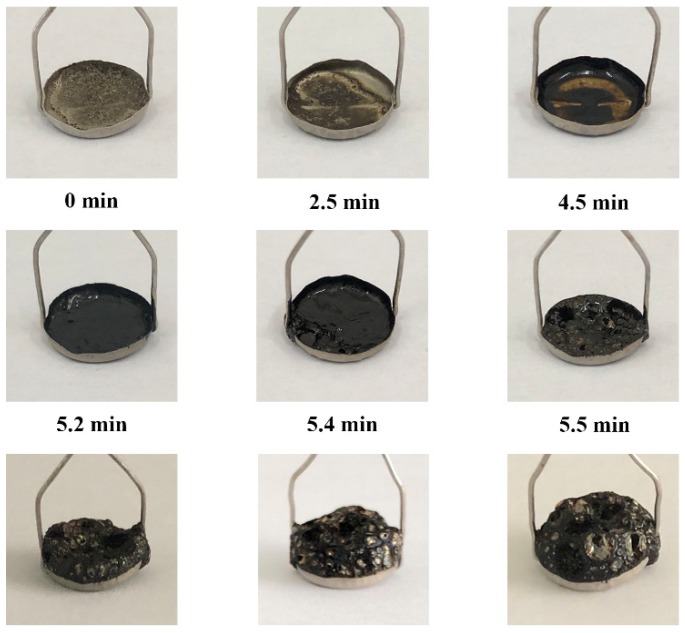
Images of sodium phenolates with time at 495 °C.

Figure 1 shows the images of sodium phenolate with time at 495 °C. Virgin sodium phenolate is a white powder. After it was heated to 495 °C, a very thin layer melts on the surface but the bottom layer was still white. About 2.5 min into the isothermal treatment, more molten salts were visible on the edge. At 4.5 min the sample became completely transparent and uniformly covered the bottom of the TGA pan. This suggests that reactions to generate flexible structures or species with low melting points had taken place.

Intumescence noticeably took off at 5.4 min. The visible trace of bubbles signified that a high viscosity of residue was achieved. Species with high molecular weights were generated. Afterward, the residue rapidly expanded and foaming materials covered the entire pan at 5.5 min. At and before this stage, the residues were completely soluble in methanol. This finding is interesting since it clearly establishes that the degree of carbonization is not necessarily high for intumescence to take place, which has been difficult to be clarified in the acid promoted intumescence due to significant crosslinking reactions involved in the aromatization.

Next, the residue became partially soluble in methanol and inflated considerably in volume at 5.6 min. Intumescence was nearly fully developed at 6 min. The filtered mother liquor of the methanol washed char showed only a slightly dark color, suggesting that a high degree of crosslinking or carbonization was achieved. After 6 min, the volumes of intumescent chars became little changed. The mother liquor of the methanol washed char was colorless and the intumescent process was deemed to be complete for the purpose of this study.

#### 3.1.2. FTIR and NMR Studies of Residues

To gain structural changes during the intumescence, characterizations by FTIR and NMR techniques of soluble portions of isothermally treated samples were carried out.

**Figure 2:**
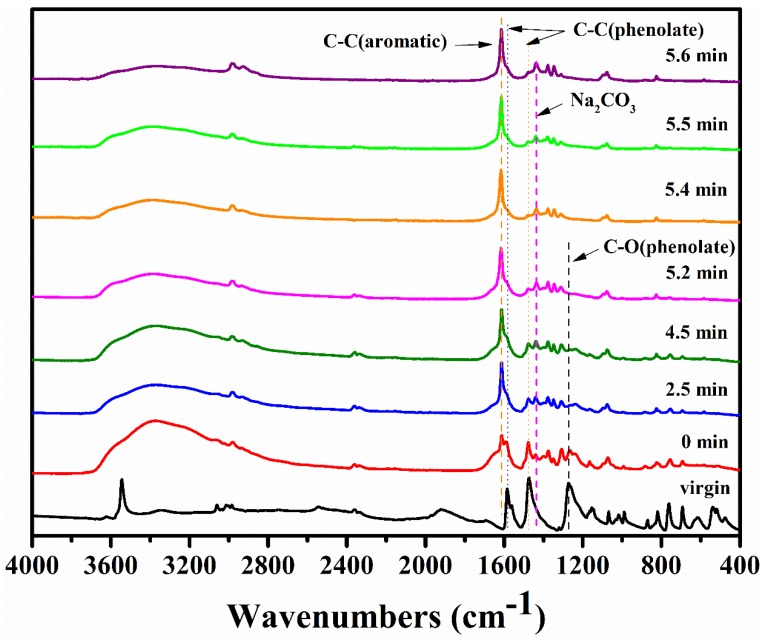
FTIR spectra of sodium phenolate (soluble part) with time at 495 °C.

First, the FTIR spectra as shown in Figure 2 clearly displayed two noticeable changes in the absorptions of benzene ring stretch at 1584 cm^−1^ and 1472 cm^−1^ and C–O stretch at 1273 cm^−1^ [30,31]. The intensities of all of these peaks decreased dramatically with time and they nearly disappeared at 4.5 min. The decreased absorptions of benzene ring stretch suggest that the NaO-substituted benzene ring is transformed to other structures while the disappearance of the C–O stretch implies a disconnect of oxygen from the benzene ring. In fact, two new peaks at 1615 cm^−1^ and 1438 cm^−1^ respectively appeared and dominated at 5.6 min. Apparently, the peak at 1615 cm^−1^ arose from aromatic structures derived from the benzene ring. On the other hand, the peak at 1438 cm^−1^ was identified to be the absorption of Na_2_CO_3_ (from neutralization of NaOH by CO_2_ in air) [22]. Thus, reactions involving the benzene ring and the cleavage of the C–O bond were essentially responsible for the changes observed in the IR spectra.

**Scheme 1:**
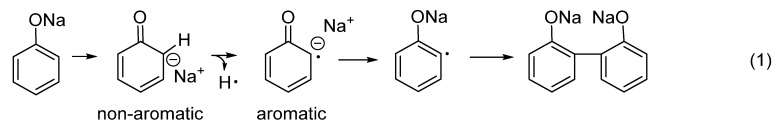
Cleavage of Ar-H bond and generation of biphenolates.

**Figure 3:**
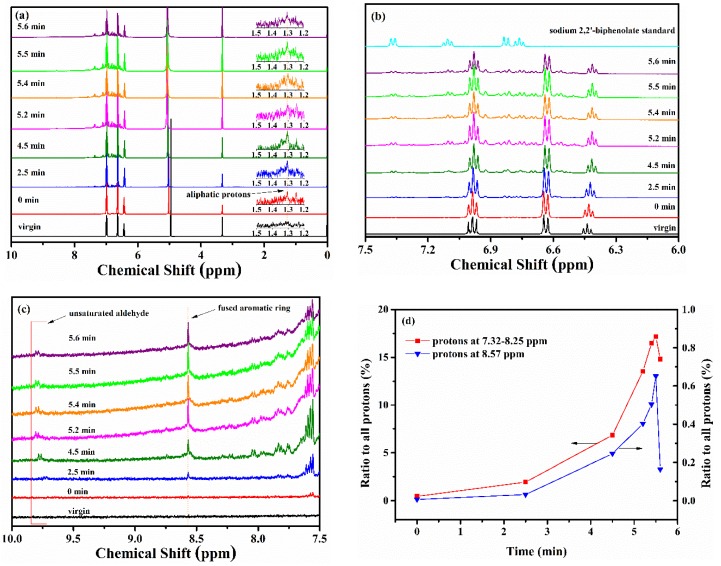
^1^H NMR spectra of sodium phenolates with time at 495 °C. (**a**): whole spectra; (**b**): aromatic protons at 6.0–7.5 ppm; (**c**): PA, PAH, and aldehyde protons at 7.5–10.0 ppm; and (**d**): ratios of PA and PAH protons to all protons.

Second, highly useful information concerning structural changes was also obtained from the ^1^H NMR analysis of aromatic protons. For all heated samples, many new doublet and triplet peaks appeared in the aromatic region. On the basis of the chemical shift of benzene proton in CD_3_OD at 7.33 ppm, most peaks below 7.33 ppm simply arose from oxygenated aromatic ring protons while those with chemical shifts larger than 7.32 ppm likely originated from protons of PAH and polyarenes (PA) [32,33]. As a matter of fact, peaks at 8.50–8.60 ppm are strong indications that fused aromatic rings have been formed [32,33,34,35]. In addition, 2,2′-biphenolate as one of the simplest substituted polyarenes was identified as one of the major products in the heated samples. Its presence is confirmed by comparing with an authentic sample (see Figure 3b). The generation of biphenolate was established to result from the combination of two aryl radicals as shown in Reaction (1) (to see Scheme 1).

It is noted that the peak area ratio of (PA + PAH) at 7.32–8.25 ppm to all protons (AP) increased exponentially until the beginning of intumescence and reached a value of 0.17 at 5.5 min as shown in Figure 3d. Concurrently, the peak area ratio of protons at 8.50–8.60 ppm to all protons multiplied. These results signified that a huge amount of (PA + PAH) was rapidly produced from the benzene ring. As a result, the degraded materials contained abundant polyaromatic stacks that subsequently lead to the intumescence at 5.5 min. Shortly after the intumescence, the ratios decreased due to the formation of insoluble matters.

Information about the aliphatic structures was also obtained from the ^1^H NMR spectra. There were new peaks at 1.12–1.39 ppm. However, the intensities of these peaks were surprisingly low. The ratio of aliphatic protons to all protons (Al/AP) was exceedingly small, and never exceeded a value of 0.002 until the beginning of the intumescence. This low value casts serious doubt on the role of aliphatic structures as a necessary soft segment to adjust the viscosity of degraded materials in the base promoted intumescence of phenols.

Besides peaks from aromatic and trivial aliphatic protons, there are two small but sharp singlets at 2.19 and 1.91 ppm respectively. Their origins remained to be elucidated. Interestingly, a doublet with a coupling constant of 8.07 Hz appeared at 9.77 ppm. This doublet is assigned to an unsaturated aldehyde proton [32]. The presence of the aldehyde group suggests that the cleavage of the benzene ring must take place, albeit at a low degree.

**Figure 4:**
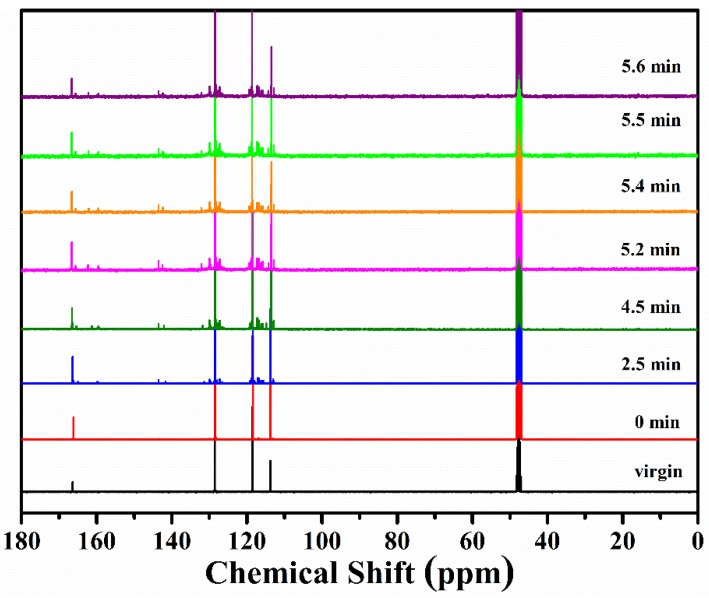
^13^C composite pulse decoupled (CPD) NMR spectra of sodium phenolates with time at 495 °C.

Further, the ^13^C NMR spectra of the residue were analyzed to examine structural changes. It could be seen that many new aromatic carbons appeared at 113–136, 141–144, and 159–169 ppm as illustrated in Figure 4. Considering the chemical shifts and ^1^H NMR and IR results, new peaks at 129–135 ppm can be assigned to fused aromatic ring carbons [32,36], those at 141–144 ppm probably arose from aryl substituted carbons of polyarenes [37], and peaks at 159–169 ppm are due to oxygen substituted aromatic carbons [32]. Consistent with ^1^H NMR, no significant signals associated with aliphatic carbons could be identified in ^13^C NMR. Thus, the importance of aliphatic structures could be ruled out in the base promoted intumescence of phenols.

#### 3.1.3. Molecular Weights of Degraded Materials

**Figure 5:**
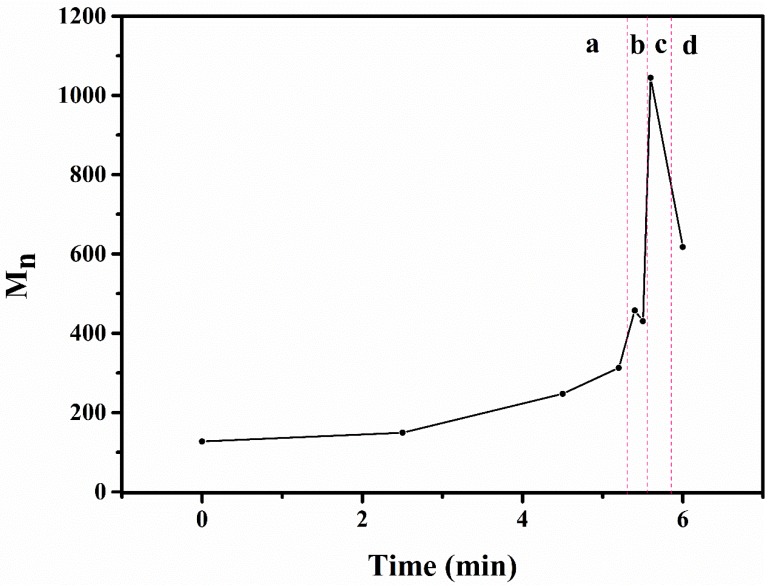
Number-average molecular weight of thermally treated sodium phenolate.

The number-average molecular weights (M_n_) of degraded materials were measured by GPC and are shown in Figure 5. It can be seen that the molecular weight increased slowly in the first five minutes of isothermal treatment. However, the molecular weight soared thereafter and reached more than 1000 Da. in 1.5 min. This is in line with the fast formation of intumescence as shown in Figure 1. After 6 min, the residues became crosslinked and the black organic portions were sparsely soluble in the solvent. Consequently, molecular weights of soluble organic residues decreased sharply. From GPC results, it could be clearly seen that the molecular weights of residues before and at the beginning of intumescence were relatively small. As a result, the generated aromatic species were most likely meltable without aliphatic moiety [38]. Aliphatic structures have been deemed to be important in the regulation of the viscosity in the acid promoted intumescence [19]. However, in view of extremely small quantities of aliphatic structures and relatively low molecular weights, the meltable aromatic structures alone must be sufficient for achieving an appropriate viscosity needed for the base promoted intumescence of phenols.

Stage a: not intumescent; stage b: intumescent and completely soluble in methanol; stage c: intumescent and partially soluble in methanol; and stage d: sparsely soluble.

**Figure 6:**
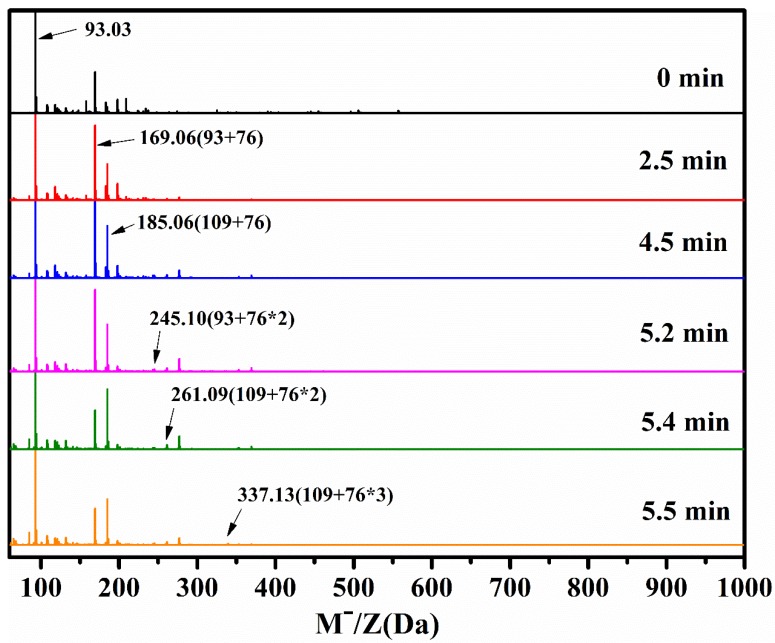
Q-TOF spectra of sodium phenolates with time at 495 °C.

To check the pattern of the molecular weights, residues were further analyzed by Q-TOF as shown in Figure 6. Visually, the molecular weights were small too. Some most discernible peaks were those with molecular weights (MW) of 93 + 76n (n = 0, 1, 2) and 109 + 76n (n = 0, 1, 2, 3, 4). These patterns suggest that both series of molecules involve the continuous buildup of phenylene group in the structure and that two series only differ by one oxygen.

**Scheme 2:**
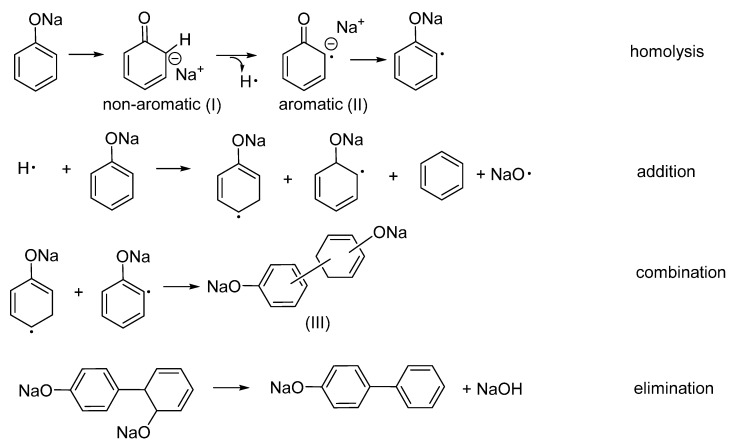
Hydroarylation and elimination of sodium phenolate.

To account for the buildup of phenylene in molecules, a four-step reaction involving homolysis, addition, combination, and elimination was proposed in Scheme 2. The homolysis step is shown in Reaction (1), which produces a hydrogen radical and aryl radical. Hydrogen radical adds to the benzene ring of the second phenolate to generate allyl radical or NaO· and benzene, which works as a blowing agent [22]. Allyl radical subsequently reacts with another aryl radical to yield a dimer (III) of sodium phenolate. The dimer is facilely transformed to phenylphenolate through the elimination of NaOH to regain aromaticity. The overall result is a hydroarylation reaction followed by an elimination step. Similarly, polyphenylenes can be generated through further hydroarylation and elimination on phenylphenolate by sodium phenolate.

**Scheme 3:**
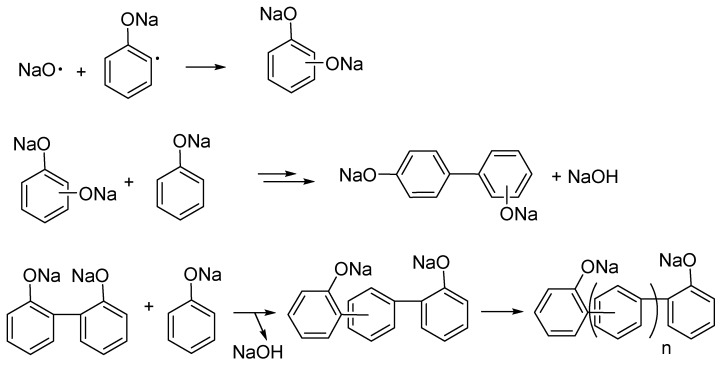
Generation of molecules with MW = 109 + 76n.

For the series of molecules with MW = 109 + 76n (n = 0, 1, 2, 3, 4), two different approaches can be devised. The first one involves biphenolate as the starting molecule while the second requires di-oxygenated benzene. Biphenolate is formed by Reaction (1) while di-oxygenated benzene is produced from the combination of aryl radical and NaO· as shown in Scheme 3. Hydroarylation of biphenolate or di-oxygenated benzene by phenolate followed by elimination reactions readily yields molecules with MW = 109 + 76n.

**Scheme 4:**
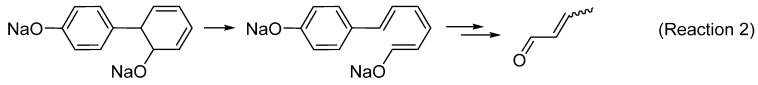
Generation of unsaturated aldehydes.

The above schemes not only justify the difference of molecular weights by 76 and 16 respectively, but also readily accounts for the disconnect of oxygen from the benzene ring and clarifies the disappearance of the C–O stretch in the IR spectra. Additionally, they rationalize the presence of peaks at 141–143 ppm, which are due to polyphenylenes, in the ^13^C NMR spectra. What is more, cyclohexadiene moiety can undergo a reverse electrocyclic reaction to triene, which subsequently rearranges to unsaturated aldehyde as shown in Scheme 4. This explains the cleavage of original benzene ring and the presence of a doublet at 9.77 ppm in the ^1^H NMR.

**Scheme 5:**
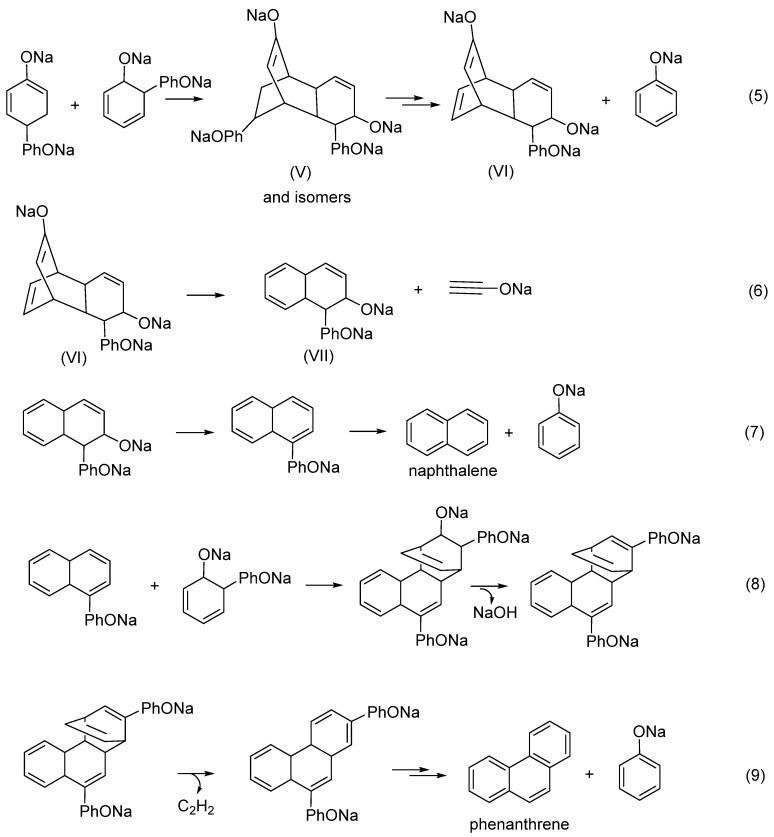
Exemplary pathways to PAHs from dimer of sodium phenolate.

Further, the cyclohexadiene structures in dimer are subjected to cyclization reactions. For those dienes with NaO-attached on an unsaturated carbon, they have especially high reactivity to participate in the Diels–Alder reaction owing to the increased electron density from the oxygen anion. The cyclization reaction provides a rapid access to fused aromatic rings. Scheme 5 shows exemplary pathways to PAHs from sodium phenolate.

First, a Diels–Alder reaction of diene produces bicyclo[2,2,2] (V). (V) is a substituted phenolate in which the bond of the ^-^OAr-substituent is activated for homolysis. The homolytic cleavage of the ^-^OAr-substituent followed by a hydrogen abstraction easily produces (VI). (VI) extrudes an alkyne to (VII). Alkyne can further cyclize with cyclohexadienes (III) to polyarenes. For (VII), it keeps on eliminating sodium phenolate and sodium hydroxide, and ultimately is converted to naphthalene. Following similar paths, larger PAHs are generated from further cyclization and elimination reactions. An example for generating phenanthrene is illustrated in Scheme 5. Thus, fused aromatic rings are promptly produced.

From the above results, it becomes clear that PAHs and polyphenylenes have the same precursor, i.e., dimer of sodium phenolate. This explains their simultaneous appearance in the ^1^H NMR. Since PAHs and polyphenylenes are also major products identified in the NMR spectra, they must be critical to the intumescence. In this sense, the generation of hydrogen radical and the subsequent hydroarylation are the two most important steps since they generate dimer. In addition, hydrogen radical attacks *ipso*-carbon to yield benzene, which works as a blowing agent [22]. Further, since PAHs and polyphenylenes have relatively low molecular weights, they are meltable [38]. This feature rules out the need of aliphatic structures to participate in the intumescent process of phenols, on contrary to the acid promoted intumescence. Overall, the generation of a hydrogen radical is the basis for the base promoted intumescence of phenols.

### 3.2. Substituents’ Effect on the Intumescence

Considering that most decomposition products of oxygenated heavily aromatic polymers are substituted phenols [39,40], a series of sodium phenolates with various substituents on benzene ring were subsequently investigated in order to examine the substituent’s effect on the intumescence.

**Figure 7:**
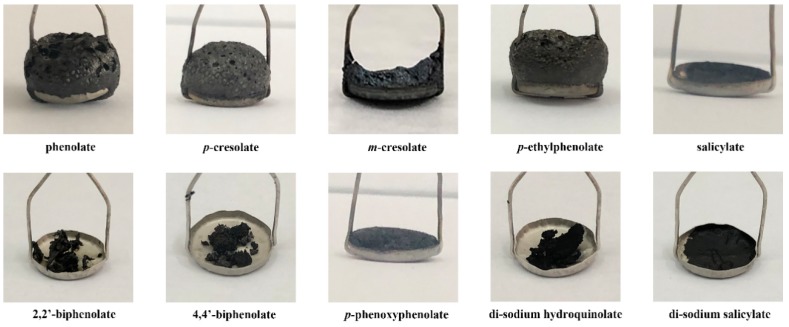
Images of char residues of sodium phenolates collected at 600 °C.

Figure 7 shows images of char residues of substituted phenolates collected at 600 °C. Notably, these substituted phenolates demonstrate a vast difference in their ability to form intumescence. Sodium salts of phenol, *p*-cresol, *m*-cresol, and *p*-ethylphenol intumesce remarkably while *p*-phenoxyphenolate, 4,4′-biphenolate, 2,2′-biphenolate, and hydroquinolate, and mono-/di- sodium salicylate do not form expanded carbonaceous char at all.

**Scheme 6:**
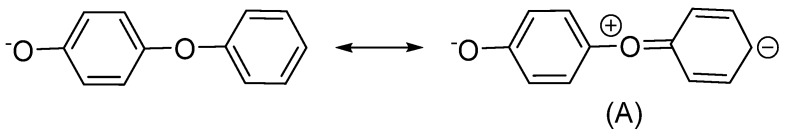
Electron withdrawing effect of phenoxy group.

A close examination of the relationship between substituents and the intumescence points that hydrogen or a weak electron donating group (EDG-W) enables intumescence but an electron withdrawing group (EWG) or a strong electron donating group (EDG-S) suppresses intumescence. The former includes alkyl groups and the latter comprises of phenoxyl (EWG), –O^−^, –PhO^−^, and –C(O)O^−^ (EDG-S). The electronic nature of the phenoxy group might be dubious, but its function as an EWG can be readily justified by the resonant structure (A) with a positive charge on the bridging oxygen (to see Scheme 6).

**Scheme 7:**
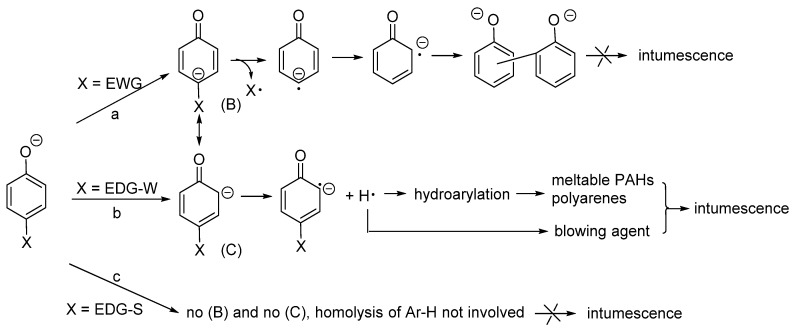
Substituent effects on the intumescence.

Since intumescence requires the participation of PAHs and polyarenes, the effect of substitutes on the intumescence can be rationalized by the easiness of the generation of hydrogen radical and the subsequent hydroarylation, which produces PAHs and polyarenes. Apparently, when the substituent X is EWG, keto form (B) is preferred over (C) as shown in route (a) in Scheme 7. As a result, the homolytic cleavage of the Ar-X to X radical prevails. There is no hydrogen radical produced in this route. The final product is XH and biphenolates. *p*-Phenoxyphenolate likely belongs to this instance. On the other hand, when X is EDG-W, keto form (C) has a lower energy than keto form (B). Accordingly, a hydrogen radical is generated, leading to both gaseous products as blowing agents and hydroarylation, which eventually produces meltable PAHs and polyarenes. Intumescence subsequently develops. This is the case for alkyl substituted phenolates. Route (c) is for X = EDG-S. Due to the presence of negative charge on the second substituent, tautomerization of phenolates to keto forms (B) and (C) is difficult to happen due to the electron–electron repulsion. Thus, Ar–H is not activated for homolysis. This might be responsible for the incapability of salicylates and biphenolates to form intumescence.

To support the above postulation, the thermal degradations of *p*-cresolate, *p*-phenoxyphenolate, salicylate, and hydroquinolate were performed in a continuously heated mode and their solid products have been carefully analyzed.

#### 3.2.1. Sodium Alkylphenolates

**Figure 8:**
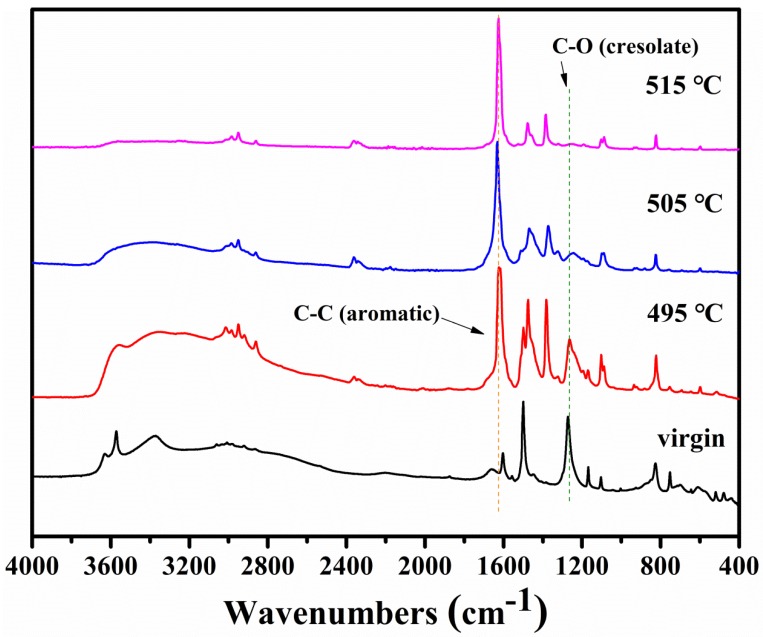
FTIR spectra of sodium *p*-cresolate (soluble part) with temperature.

The FTIR spectra of sodium *p*-cresolate with temperature are shown in Figure 8. The obvious change is the gradual disappearance of the C–O stretch at 1263 cm^−1^. This is similar to what happens to sodium phenolate and indicates C–O bond scission. [31] The disconnect of the C–O bond in the thermal degradation of phenolate involves a hydrogen radical. Sodium *p*-cresolate probably undergoes a similar homolytic cleavage of the Ar–H bond during its thermal degradation.

**Figure 9:**
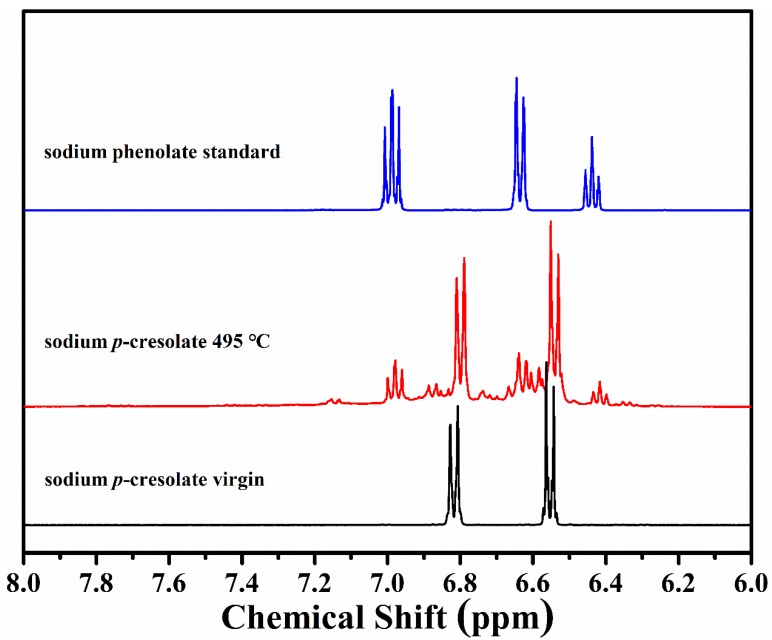
^1^H NMR spectra of sodium *p*-cresolate collected at 495 °C.

What is more, ^1^H NMR of the residue collected at 495 °C demonstrated the presence of sodium phenolate as shown in Figure 9. This suggests that the reduction of *p*-cresolate by hydrogen radical, generated from route (b), took place. Since no biphenolates can be identified in the ^1^H NMR, route (a) was not important for the thermal degradation of *p*-cresolate.

**Figure 10:**
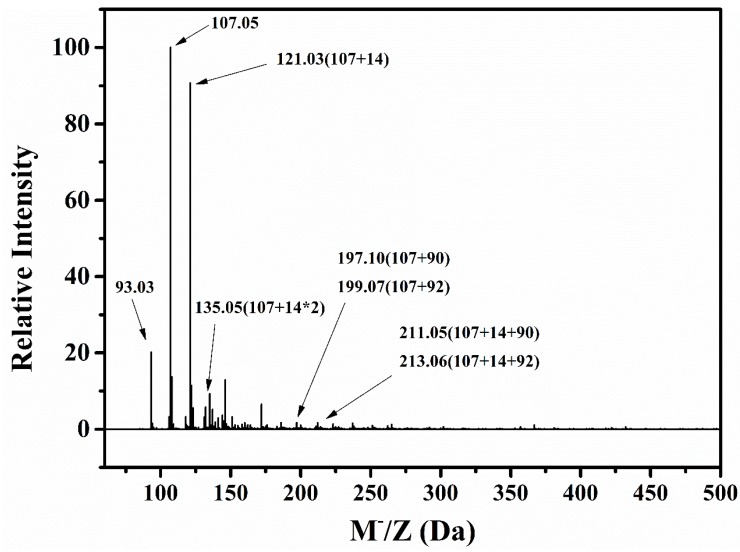
Q-TOF spectrum of sodium *p*-cresolate collected at 495 °C.

**Scheme 8:**
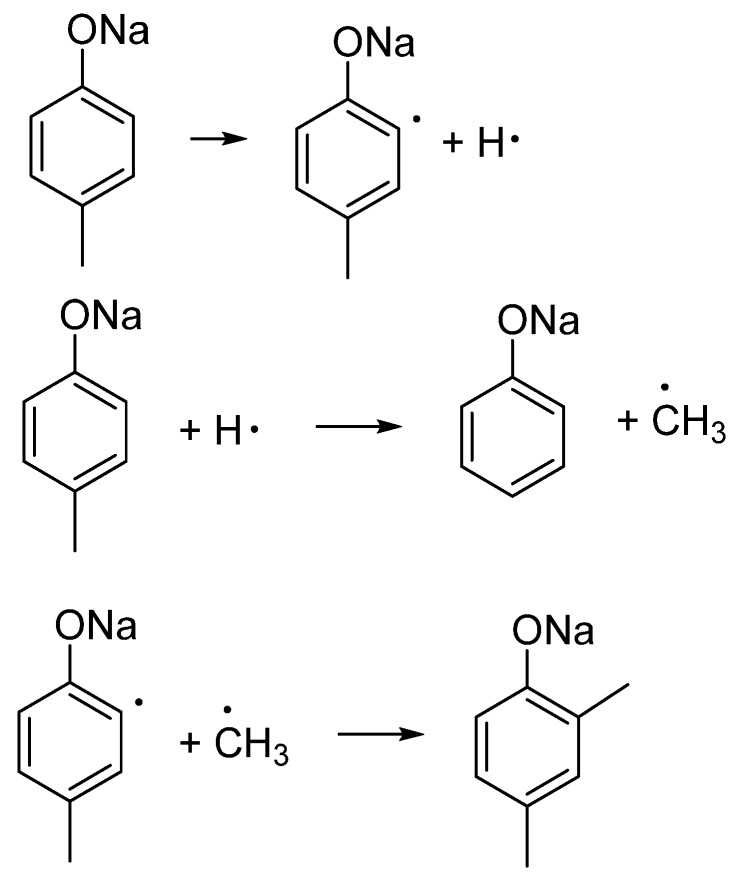
Scrambling reaction of *p*-cresolate to phenolate and multi-methyl substituted phenolates.

**Scheme 9:**
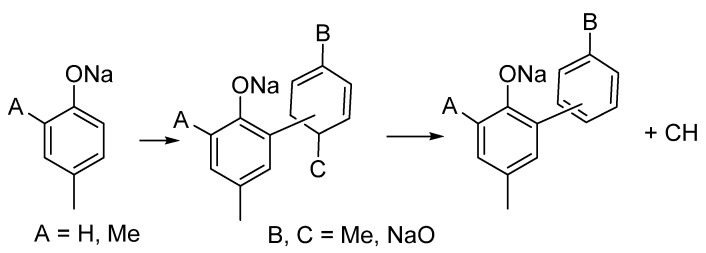
Formation of polyarenes from *p*-cresolate.

The generation of hydrogen radical was further supported by the Q-TOF analysis. Peaks with a molecular weight of 93, 107 + 14n (n = 0, 1, 2), 107 + 14n + 92m (n = 0, 1, m = 1)), and 107 + 14n + 90x (n = 0, 1, x = 1)) show up as seen in Figure 10. Evidently, the peak of 93 is from phenolate while those with MW = 107 + 14n (n=0, 1, 2) arose from multi-methyl substituted phenols, a result from scrambling reaction between two *p*-cresolates via hydrogen radical as illustrated in Scheme 8. The difference of molecular weight by 90 or 92 indicates the presence of the methylphenyl or hydroxyphenyl group. These structures are formed by hydroarylation followed by the elimination of either methane or NaOH as illustrated in Scheme 9. As a consequence of hydroarylation, intumescence of *p*-cresolate eventually takes place.

**Scheme 10:**
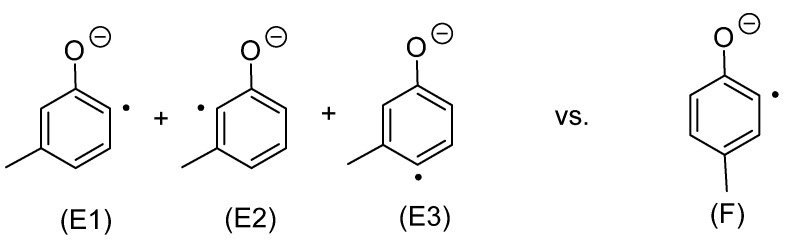
Steric effect of substituents on the intumescence.

The intumescent process of *p*-ethylphenolate is analogous to that of phenolate and *p*-cresolate. All of them intumesce strongly. However, *m*-cresolate is somewhat different. It intumesces much less than *p*-cresolate as seen in Figure 7. This difference is probably caused by the electronic effect of *o*-, *p*-, and *m*- alkyl groups on the activation degree of the Ar–H bond. Nevertheless, a steric effect might be important as well. Since the generation of dimer relies on radical combinations, it is not inconceivable that while E1 has a comparable reactivity as F (to see Scheme 10), both E2 and E3 have a reduced reactivity toward radical couplings owing to the steric congestion introduced by the neighboring methyl group. Subsequently, hydroarylation is restricted resulting in reduced intumescence of *m*-cresolate.

#### 3.2.2. Sodium *p*-Phenoxyphenolate 

**Figure 11:**
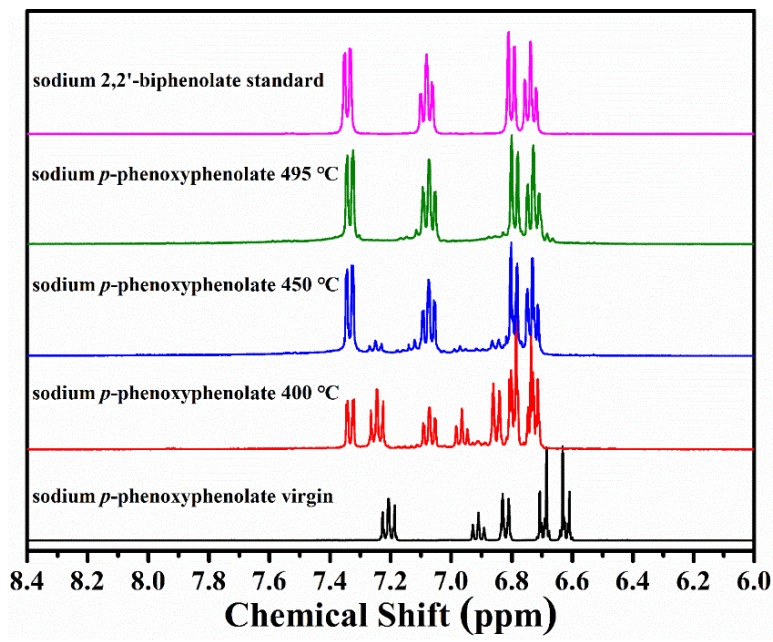
^1^H NMR spectra of thermally treated sodium *p*-phenoxyphenolate at different temperatures.

The thermal degradation of sodium *p*-phenoxyphenolate produces phenol and 2,2′-biphenolate as major products. These products are identified by TGA-FTIR [22] and ^1^H NMR. The ^1^H NMR spectrum of the residue collected at 495 °C is identical to those of sodium 2,2′-biphenolate as shown in Figure 11. The preferred formation of 2,2′-biphenol instead of 4,4′-biphenol suggests that the bisallyl carbanion radical has a higher energy than the *o*-carbanion radical as shown in route (a). Clearly, the thermal degradation of *p*-phenoxyphenolate takes route (a) in Scheme 4.

#### 3.2.3. Biphenolates and Sodium Salicylates

Both 4,4′- and 2,2′-biphenolates are thermally stable up to 550 °C, after which only a continuous mass loss at a very slow rate was observed. Apparently, Ar–H in biphenolates is not activated by an oxygen anion as those in phenolate or alkylphenolates. Otherwise, a rapid weight loss should be observed.

**Figure 12:**
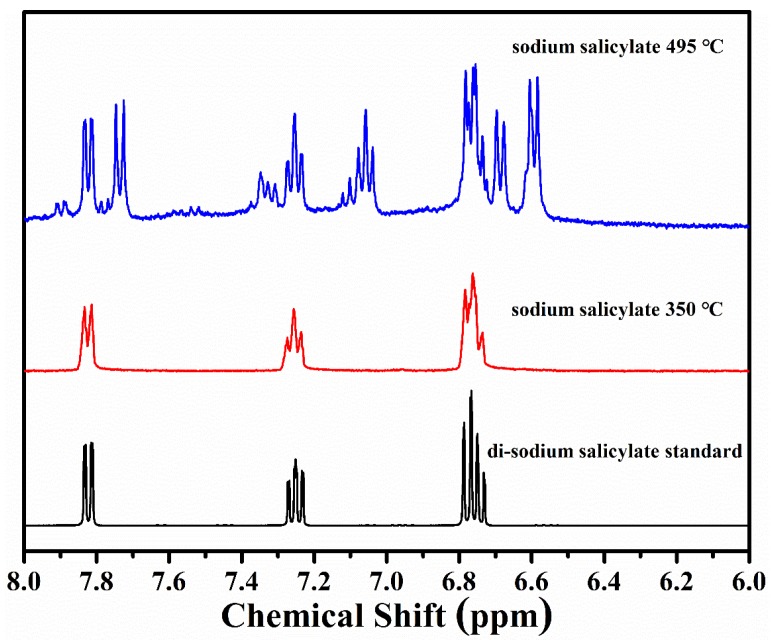
^1^H NMR spectra of thermally treated sodium salicylate.

**Figure 13:**
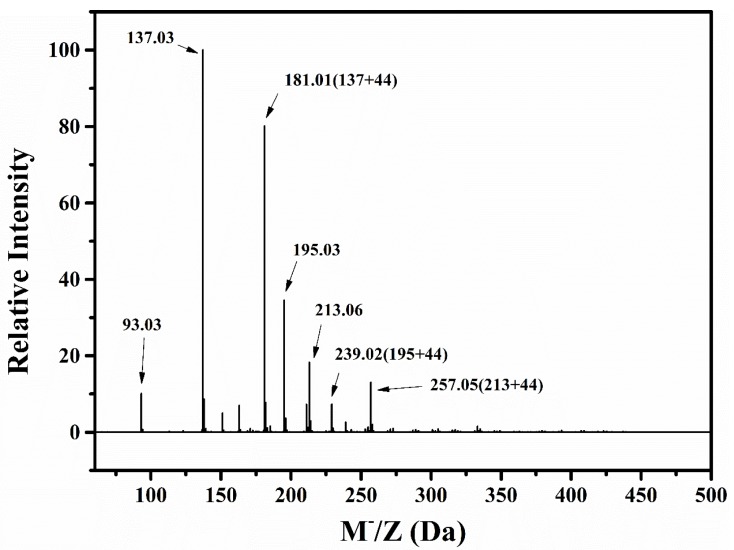
Q-TOF spectrum of sodium salicylate collected at 495 °C.

Mono-sodium salicylate was thermally converted to di-sodium salicylate below 350 °C. The ^1^H NMR of the residue collected at 350 °C matched that of di-sodium salicylate. Although di-sodium salicylate underwent thermal degradation beginning at 450 °C with a mass loss of 14.2%, the ^1^H NMR pattern of the residue collected at 495 °C revealed that it mainly contained 1,2-di-substituted benzenes as shown in Figure 12. No 2,2′-biphenol could be identified and hence route (a) was not viable. Instead, 1,2-di-subsitituted benzenes were largely derived from the esterification reaction and decarboxylation. The Q-TOF result (to see Figure 13) shows peaks with molecular weights = 93 (phenolate), 137 (salicylate), 137 + 44, 195, 195 + 44 (cyclic diester), 213 (phenyl salicylate), and 213 + 44. The difference by 44 in MW suggests decarboxylation or carboxylation, which did not involve a free radical. Therefore, Ar–H in salicylate was not activated by an oxygen anion either.

## 4. Conclusions

The intumescent process of substituted phenolates was studied. The generation of hydrogen radical was the most critical step. It led to the production of blowing agents and hydroarylation. Hydroarylation yielded reactive cyclohexadienes from which both polyarenes and PAH were facilely generated through a series of elimination and cyclization reactions. Polyarenes and PAH had relatively low molecular weights and hence were meltable. This feature called for no aliphatic structures in the base promoted intumescence of phenols.

The substituents showed great influence on the intumescent ability of phenolates. Phenolates substituted with weak EDG enabled intumescence while those with EWG or strong EDG did not form expanded carbonaceous chars. This discrete outcome could be justified by both the electronic effect of substituents, which dictated the easiness of the generation of hydrogen radical and the steric factor, which controlled the degree of hydroarylation.
